# Supplementary material for: Knowledge-based prediction of protein backbone conformation using a structural alphabet
Source: PLoS One. 2017 Nov 21;12(11):e0186215. doi: 10.1371/journal.pone.0186215 (PMC5697859; doi:10.1371/journal.pone.0186215)
Supplement: S1 File — (PDF) [file pone.0186215.s001.pdf]

SUPPORTING INFORMATION

Knowledge based prediction of protein backbone conformation using a structural alphabet

Iyanar Vetrivel, Swapnil Mahajan, Manoj Tyagi, Lionel Hoffmann, Yves-Henri Sanejouand, Narayanaswamy Srinivasan, Alexandre G. de Brevern, Frédéric Cadet, Bernard Offmann

Table A. Distribution of total penta-PBs and unique penta-PBs at different sequence identity cut-offs in the PDB.

Seq. identity cut-off (%)	Total number of penta-PBs	Total number of unique penta-PBs
30	4,366,040	40,130
40	5,239,216	42,779
50	6,025,703	45,182
70	7,053,751	48,186
90	7,895,276	50,597
95	8,229,825	51,514
100	12,508,483	57,889
**Full PDB**	**61,208,019**	**94,263**

Table B. Assessment of the list of PBs reported after querying PB-PENTAdb for the 15,544 query proteins from PDB30 datatset. Shown is the number of times the correct PB were in the list of all possible PBs.

Protein Block	Total	Correct PB in list	Percentage
a	145,102	96,501	66,5%
b	172,135	86,256	50,1%
c	332,295	207,031	62,3%
d	753,725	573,645	76,1%
e	84,282	48,114	57,1%
f	257,369	156,029	60,6%
g	45,250	18,251	40,3%
h	83,152	48,627	58,5%
i	58,657	31,993	54,5%
j	32,598	17,356	53,2%
k	209,416	131,400	62,7%
l	207,772	127,305	61,3%
m	1,294,063	1,119,179	86,5%
n	69,954	41,171	58,9%
o	95,682	59,422	62,1%
p	130,506	74,971	57,4%
**Total**	**3,826,856**	**2,740,750**	**71,6%**

Table C. Confusion matrix for the predictions by Hybrid method using *noise filtering scheme* for querying PENTAdb.
The PBs in the column are the actual PBs and the ones in the rows are the predictions. The diagonals are the raw counts for the correctly predicted PBs and these served for calculating the PB-wise identities and the overall accuracy of PB-kPRED (see also Table 4). The off-diagonal values highlighted in grey are the confused predictions that are still acceptable for they correspond to PB substitutions frequently observed among homologous preoteins (Tyagi et al. 2006; Joseph, Srinivasan, and De Brevern 2011). These served for calculating the PB-wise similarities and the overall Q16 similarity. The last column (Δ) represents the PB-wise difference between similarity and identity. It highlights those PB-wise predictions which gained upon considering similarity as a criteria for evaluating the predictions.

	a	***b***	***c***	***d***	***e***	***f***	***g***	***h***	***i***	***j***	***k***	***l***	***m***	***n***	***o***	***p***	Identity	Similarity	Δ
a	97,530	3,235	8,333	8,735	856	3,307	1,125	951	1,108	640	3,010	3,013	9,290	608	1,331	2,063	67.20%	72.94%	5.74%
b	4,388	89,835	9,249	13,708	1,797	5,395	1,196	4,246	2,175	794	7,254	9,615	14,759	1,122	3,195	3,535	52.15%	55.88%	3.73%
c	11,022	8,894	194,601	30,899	2,640	11,788	3,639	3,113	1,849	1,592	7,000	6,465	34,343	1,804	4,605	8,226	58.53%	62.94%	4.41%
d	14,121	19,601	42,215	505,429	10,584	26,320	3,788	9,320	5,167	3,805	14,682	13,552	67,308	3,622	5,949	8,887	67.00%	67.00%	0.00%
e	1,167	1,631	2,201	6,703	48,449	4,745	2,928	1,861	661	664	3,128	1,273	4,765	2,913	551	689	57.45%	68.76%	11.31%
f	4,518	6,082	11,838	19,794	5,775	155,294	2,192	3,496	1,737	1,782	8,398	7,433	22,369	1,897	1,920	2,990	60.30%	63.24%	2.93%
g	1,251	942	2,841	2,139	2,499	1,602	19,673	492	348	277	1,177	1,152	5,401	2,387	1,037	2,061	43.45%	67.36%	23.91%
h	1,282	2,687	2,126	4,031	1,437	2,122	559	50,780	874	727	3,424	2,377	4,375	626	5,003	751	61.05%	71.00%	9.95%
i	1,362	1,685	1,575	2,648	407	1,279	340	760	34,715	2,090	1,224	1,830	3,076	378	623	4,674	59.17%	73.58%	14.40%
j	792	501	1,016	1,700	460	1,220	256	842	2,703	16,307	846	899	1,663	279	365	2,777	49.98%	67.18%	17.20%
k	3,927	5,781	6,710	9,723	2,864	6,944	1,571	4,461	2,129	1,752	133,967	6,028	16,908	2,183	2,161	2,429	63.93%	66.90%	2.97%
l	4,767	8,735	7,073	9,661	1,301	7,174	1,390	2,711	2,668	1,641	6,150	124,727	21,404	1,982	3,569	2,969	59.99%	60.78%	0.79%
m	17,605	20,513	41,891	69,175	6,385	26,869	8,090	7,242	4,910	4,379	25,529	26,605	982,610	14,685	17,029	21,327	75.89%	75.89%	0.00%
n	843	906	1,456	1,993	2,394	1,286	2,024	541	364	352	1,821	1,668	8,402	43,511	1,107	1,343	62.15%	68.54%	6.39%
o	1,588	2,265	2,939	2,904	411	1,344	790	4,830	622	365	1,573	2,788	9,575	893	60,484	2,346	63.19%	64.95%	1.76%
p	2,365	2,982	5,814	5,445	563	2,274	1,627	740	4,406	2,531	2,137	2,261	16,615	1,575	1,880	77,334	59.24%	65.07%	5.83%
																Average Q16	66.31%	68.85%	

Table D. Comparison of kPRED to secondary structure assignment from dictionary of protein secondary structure (DSSP)

Sequence identity cut-off values	α-helix/3_{10} helix/π-helix mapped to PBs *k/l/m/n/o* and extended *β*-strand mapped to PBs *a/b/c/d/e/f*	α-helix/3_{10} helix/π-helix mapped to PBs *l/m/n* and extended *β*-strand mapped to PBs *c/d/e*
100 %	80.94 %	70.30 %
<100 %	78.81 %	67.96 %
<95 %	78.81 %	67.96 %
<90 %	78.81 %	67.96 %
<70 %	78.81 %	67.96 %
<50 %	78.81 %	67.96 %
<40 %	78.80 %	67.96 %
<30 %	65.90 %	55.20 %

Shown above are the results of comparing the 16 state PB prediction from kPRED to the reduced 3 state secondary structure assignment by DSSP (Kabsch and Sander, 1983). PBs *k, l, m, n, o* were related to helical repetitive structures (3_10, alpha and pi helices) while *PBs a, b, c, d, e, f* were related to extended structures (beta strands). These PBs correspond to central part of these repetitive structures and to their capping regions as shown by de Brevern (de Brevern, 2000). The kPRED algorithm could be predicting sub-secondary structure details for example, kinks and bends in helices by PB other than *k,l,m,n,o* but the three state accuracy (Q3) will favor us negatively in these cases. Hence measuring the performance of kPRED by Q3 accuracy is not totally justified because kpred algorithm was not designed with the objective of secondary structure prediction in mind. Interestingly, this mapping of kPRED prediction to SS status shows that kPRED accuracy (80.94%) is largely better than the prediction accuracy of (i) 66.4% of the method by Zhang et. al., which does not use evolutionary information (J. Mol. Biol. 225, 1049-1063) and (ii) 76.5% of the PSI-PRED method which uses evolutionary information (Jones, D.T., 1999, J. Mol. Biol. 292, 195-202). This simple evaluation highlight the quality of kPRED, here for something not designed for it.

Fig A. Histogram depicting the distribution of prediction accuracies for 15,544 query proteins by hybrid method using noise filtering scheme when only distant homologues (<30%) are allowed in PENTAdb. The distribution is unimodal with sharp peak at the 40% accuracy range and gradually tapering towards the higher accuracies.

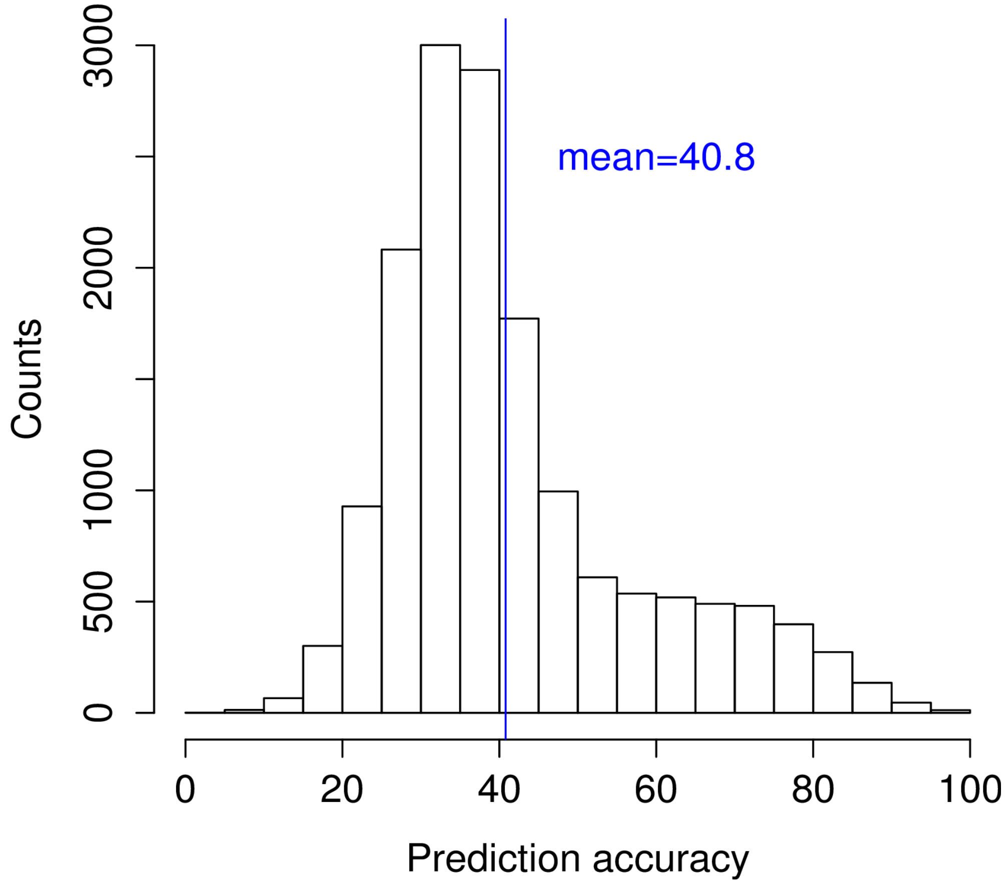

Fig B. Structure alignment of two query cases with their respective homologues. (a) Shown is the alignment of hypothetical DNA protein binding protein with its identical homologue. RMSD is 0.14Å. (b) Shown is alignment of energy-coupling factor transporter EcfT with its 2 other 100% homologues. RMSD values with query are 1.4Å and 1.9Å.

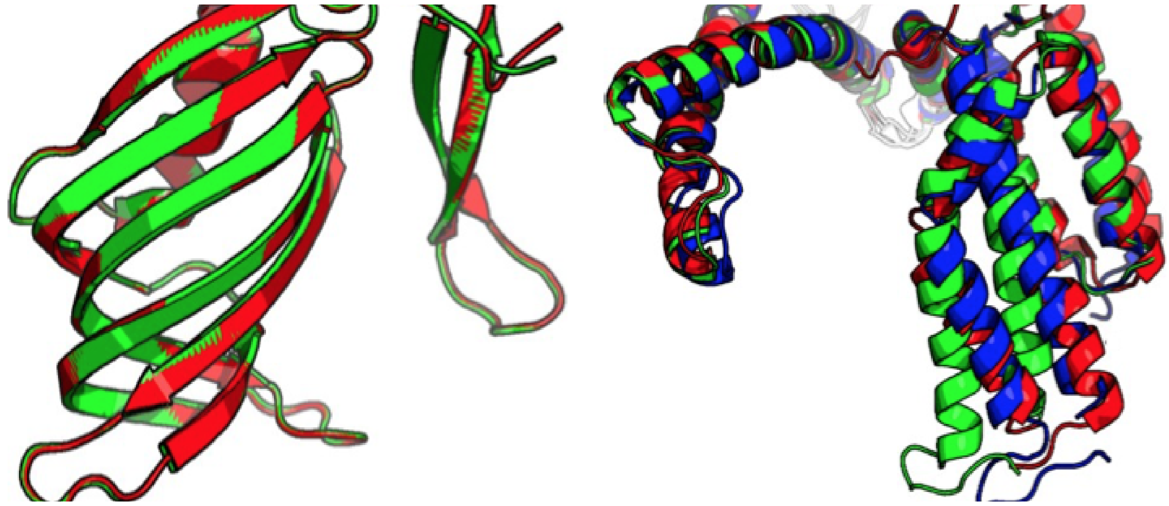

Text A. Additional method. Tri-PB and penta-PB normalized frequency tables.

The approach that was used in this work to predict the structure of pentapeptides and to evaluate the predictions relied on two frequency tables built from the PDB30 dataset. The first look-up table constituted of all theoretically possible 4,096 (16^3) tri-PB motifs and was used for predicting the structure of a pentapeptide by taking into account the structures of its immediate preceding and succeeding pentapeptides (see *Prediction scheme* section). The second look-up table is composed of all 1.04 million (16^5) possible penta-PB motifs and was used for scoring the predictions fo PB-kPRED.. The frequency of occurrence of each tri-PB and penta-PBs was normalized following equation (1) below:

$$N_{j,k} = \frac{n_{j,k}}{\prod_{i=1}^{k} p\left(PB_{i,j}\right) \cdot \sum_{j=1}^{16^k} n_{j,k}} \quad \text{(Eqn. 1)}$$

where j is an integer associated with a PB motif, k is the length of the PB motif j (*i.e* the number of successive pentapeptides) with k=3 for tri-PB motifs and k=5 for penta-PB motifs, $N_{j,k}$ is the normalized frequency of the PB motif j, $n_{j,k}$ is the occurrence (raw count) of the PB motif j in PDB30 dataset and $p(PB_{i,j})$ is the frequency of the observed PB at position i in the PB motif j.

Text B. Additional legend to Fig 6. Schemes used by PB-kPRED for querying PENTAdb

Sections of the database accessible are indicated in green and those not accessible in red. The sections are delimited by sequence identity thresholds. In the classic scheme (a), eight different experiments (A1 to A8) were performed. They differ by the sections of PENTAdb that were made accessible for every pentapeptide query. For each of the 15,544 query protein sequences, the portion of PENTAdb accessible for prediction was dynamically determined using MySQL queries: only pentapeptides coming from protein chains in PDB that shared sequence identities below the indicated cut-off values were accessible to PB-kPRED. For example, in experiment A1, only the query was left out, the full PDB was accessible while in experiment A8, only proteins not sharing more than 30% sequence identity were made accessible for predictions. In the *noise filtering scheme*, the subset of PENTAdb that is accessible for query is dynamically expanded. Each individual vertical column represents an experiment (B1 to B8). For each experiment, a maximum homology level was used to define the subset of PENTAdb accessible by the PB-kPRED algorithm for predicting the local structure of each individual query pentapeptide. The defined maximum homology levels were ≤100%, <100%, <95%, <90%, <70%, <50%, <40% and <30% . For each query pentapeptide, data in PENTAdb only coming from closest homologues was queried first. For example, in experiments B1, B2 and B3, the highest allowed homology ranges were respectively 100%, ≥95 to <100% and ≥90 to <95% . If hits were found, these were used to constitute the list of all possible PBs (see text and Fig 4) and the algorithm proceeded with the next query pentapeptide. If no hits were found, subsequent subsets of PENTAdb comprising of more distant homologues were made available successively until hits were found. This is a procedure followed at an individual pentapeptide level and not at the level of the query protein sequence. Hence, in the best case scenario, this would represent a total of (l-4) queries to PENTAdb, l being the length of the sequence and in the worst case scenario, it would be 8 x (l-4) considering the 8 sequence identity cut-off levels. For the PDB30 dataset, this amounts to a total of 3.97 million queries in the best case scenario and up to 31.76 million queries in the worst case scenario.
